# The effects of chronic and acute physical activity on working memory performance in healthy participants: a systematic review with meta-analysis of randomized controlled trials

**DOI:** 10.1186/s13643-017-0514-7

**Published:** 2017-06-30

**Authors:** Azeem Rathore, Barbara Lom

**Affiliations:** 1 Department of Biology, Davidson College, Davidson, NC USA; 2 Program in Neuroscience, Davidson College, Davidson, NC USA

**Keywords:** Acute physical activity, Chronic physical activity, Healthy participants, Working memory, Cognition, Randomized controlled trials, Systematic review, Meta-analysis, Moderation analysis

## Abstract

**Background:**

Understanding how physical activity (PA) influences cognitive function in populations with cognitive impairments, such as dementia, is an increasingly studied topic yielding numerous published systematic reviews. In contrast, however, there appears to be less interest in examining associations between PA and cognition in cognitively healthy individuals. Therefore, the objective of this review was to evaluate and synthesize randomized controlled trial (RCT) studies that investigated the effects of both chronic and acute PA on working memory performance (WMP) in physically and cognitively healthy individuals.

**Methods:**

Following the preferred reporting items for systematic review and meta-analysis (PRISMA) guidelines, a systematic review of studies published between August 2009 and December 2016 was performed on RCTs investigating the effects of chronic and acute PA on WMP with healthy participants as the sample populations. Searches were conducted in Annual Reviews, ProQuest, PsycARTICLES, PsycINFO, PubMed, and Web of Science. Main inclusion criteria stipulated (1) healthy sample populations, (2) PA interventions, (3) WMP as an outcome, and (4) RCT designs. Descriptive statistics included cohort and intervention characteristics and a risk of bias assessment. Analytical statistics included meta-analyses and moderation analyses.

**Results:**

From 7345 non-duplicates, 15 studies (eight chronic PA and seven acute PA studies) met the inclusion criteria and were evaluated. Overall, there was noticeable variance between both cohort and intervention characteristics. Sample populations ranged from primary school children to retirement community members with PA ranging from cycling to yoga. The majority of studies were characterized by “low” or “unclear” risk of selection, performance, detection, attrition, reporting, or other biases. Meta-analysis of chronic PA revealed a significant, small effect size while analysis of acute PA revealed a non-significant, trivial result. Age and intensity were significant moderators while allocation concealment, blinding, and intervention length were not.

**Conclusions:**

Chronic PA can significantly improve WMP while acute PA cannot. The limiting factors for acute PA studies point to the diversity of working memory instruments utilized, unequal sample sizes between studies, and the sample age groups. Large-scale, high-quality RCTs are needed in order to provide generalizable and more powerful analysis between PA and WMP in a systematic approach.

**Electronic supplementary material:**

The online version of this article (doi:10.1186/s13643-017-0514-7) contains supplementary material, which is available to authorized users.

## Background

The effects of physical activity (PA) on various domains of cognitive function remain an ongoing and actively researched topic in cognitive psychology [1], exercise science [2], neuroscience [3], and clinical medicine [4], among other fields. The primary objectives of studies have been and continue to be the evaluation and measurement of the cognitive benefits that PA can potentially confer with special attention to cognitively impaired individuals, including those with dementia [5, 6], Alzheimer’s disease [7], Parkinson’s disease [8, 9], schizophrenia [10], or mild cognitive impairment [11, 12]. Cognition has been measured along the domains of executive function, attention, memory, and working memory, each associated with distinct psychological measurement instruments. Of note, the cognitive benefits individuals can confer largely—if not entirely—remain a function of the type of PA, namely acute PA interventions [13–15] or chronic PA interventions [16, 17]. Ergo, making definitive and strong claims regarding the effect of PA on cognitive function, is rather difficult considering the effect is moderated by various other variables, including the study design itself. For instance, moderating variables, such as the type of cognitive impairment, type of intervention, duration of PA, and perhaps most importantly, the type of population of interest, can largely explain why PA offers promising cognitive benefits in some studies [18–20] and negligible results in other studies [21, 22]. For good reason, that is why researchers employ selective and cautious language when discussing their own results, whether from a randomized controlled trial (RCT) or review, so as not to generalize potential protective effects conferred by PA beyond what the evidence shows.

However, the overwhelming consensus by healthcare officials, including the World Health Organization (WHO), remains the steadfast advocacy of PA to all persons as a means to improve quality of life metrics, such as reduction of coronary heart disease, diabetes, and hypertension [23]. Further, the WHO claims that PA can reduce cognitive decline especially in populations of 65 years and above. Such claims can easily be supported by a cursory search of the current literature. For instance, a 2006 prospective, cohort study was published that showed that regular PA was associated with a protective delay in onset of dementia and Alzheimer disease in a sample population of 65 years or older [24]. In another study, a sample population of elders 50 years or older at risk for Alzheimer’s disease were shown to have modest improvements in memory over an 18-month period when participating in physical exercise [7]. As with memory, a 2011 study showed selective benefits in executive function when examining the association between PA and an elderly population afflicted with Parkinson’s disease [9]. Perhaps unsurprisingly, then, numerous systematic reviews have been published that have offered similar conclusions about the positive effects of PA on cognition [1, 5, 8, 10, 11]. However, there also exists published studies that have concluded null associations between PA and cognition under a variety of study designs and interventions [25–27]. In fact, Cochrane, arguably the leading organization on integrating systematic reviews, meta-analyses, and interpreting medically related research, has published systematic reviews finding no significant cognitive improvement conferred by PA, including on populations afflicted with dementia [28] and populations without any known cognitive impairments [29]. Indeed, generalizing results from any individual study or review beyond the clear parameters and research question being investigated can be challenging. In other words, results showing increased cognitive function from vigorous PA in a population afflicted with dementia should not be generalized to healthy populations. An additional consideration is that despite individual studies showing statistically significant protective and beneficial effects conferred by PA on cognition, reviews with meta-analyses on the very same research question can yield null results [30]; the reasons for why such discrepancies occur will be elucidated through this review. Despite the many publications that have investigated relationships between PA and cognition as illustrated, some cognitive domains, sample populations, and study designs receive more attention than others. Intuitively, this research bias makes sense because many researchers are particularly interested in the protective effects conferred by PA on populations afflicted by some kind of cognitive impairment. For instance, individuals with chronic obstructive pulmonary disease have been a research focus for several years now through investigating different PA interventions, such as water-based exercise and resistance training [31, 32], with the underlying goal, similar to studies with cognitively impaired individuals, of identifying potential therapeutic and clinical benefits. Clearly, understating the protective effects conferred by PA continues to be a heavily focused and researched topic as evidenced both in the studies and reviews highlighted.

There remain limited studies, especially reviews, which have examined the association in healthy populations and measured working memory as an outcome. Between 2009 and 2016, for example, only one published systematic review with meta-analysis was identified that included both working memory as an outcome and healthy individuals as a population of interest [33]. In contrast, in this same time, several reviews were published that measured working memory as an outcome but among cognitively impaired populations, including those afflicted with Parkinson’s disease and schizophrenia [8, 10, 29]. Seemingly, then, the potential benefits of PA on working memory function in healthy populations has not been as rigorously studied. Thus, there appears an opportunity to review such a topic.

This review evaluated and synthesized RCTs that investigated the effects of physical activity specifically on working memory performance (WMP) in physically and cognitively healthy individuals and was warranted for several reasons. First, much of the scientific literature, and by extension systematic reviews, has focused primarily on cognitively impaired individuals with relatively less emphasis on healthy individuals. Further, this review is novel in its aim with the purpose of understanding the baseline capacity for improvements in WMP of healthy individuals, and to our knowledge, such a focused research question has not been investigated in any capacity until now. Descriptive statistics were performed to qualitatively provide summary analysis of studies while analytical analyses, including risk of bias assessment, meta-analyses, and moderator analyses, were also performed to empirically assess included studies. With regard to WMP, although several models of working memory have been proposed, this review defines working memory according to Baddeley’s definition as the “temporary storage and manipulation of information that is assumed to be necessary for a wide range of complex cognitive activities” [34]. As for the importance of working memory, researchers as far back from the 1980s identified the relation between WMP and literacy outcomes [35], and even more recently the potential correlation between WMP and sensory saliency, i.e., selective attention [36]. Thus, in more general terms, WMP has been discussed for its link with general intelligence. Finally, with respect to systematic reviews, recent evidence has indicated that despite the increased utility and popularity of this kind of format a significant proportion of published reviews are both poorly conducted and reported [37]. Therefore, this review will follow recommended practices and guidelines [38, 39] in order for maximal transparency and rigor.

## Methods

### Eligibility criteria

This systematic review was conducted according to PRISMA guidelines that aim to increase reporting and rigor by following the standardized framework recommended as well as fulfilling a 27-item checklist to ensure maximal reporting [see Additional file 1]. Therefore, the eligibility criteria were framed via another PRISMA recommendation known as the PICOS approach that pre-defines and identifies a review’s population (P), intervention (I), comparator group (C), outcome (O), and study design (S). Thus, studies were selected from the initial search if they met the following inclusion criteria:

i. Population: the sample population was identified as cognitively and physically healthy via validated diagnostic tools.
ii. Intervention: PA defined as “any bodily movement produced by skeletal muscles that result in energy expenditure” [40]. Acute PA interventions were identified as those with a single PA session while chronic PA interventions were defined as those with more than one PA session. Furthermore, PA was the purposefully selected term as it incorporates a broader spectrum of interventions that otherwise could be excluded under the term “exercise.” Thus, “physical activity” was expected to capture conventional forms of activity, such as cardiovascular exercise and resistance training, but also less conventional forms, such as yoga. Finally, no limitations were imposed based upon modality, dose, intensity, or supervision, but dual-task interventions or self-reported interventions were excluded due to confounding factors noted in previous research [33].
iii. Comparator: any kind of control group was eligible, including no treatment, waitlist, health education, sham exercise, or sedentary treatment.
iv. Outcome: validated WMP cognitive assessment tools, according to a specific categorization described below.
v. Study design: randomized controlled trials, including cluster-RCTs, crossover-RCTs that are full-length studies published in peer-reviewed, English language journals.

### Working memory tests

Working memory refers to “a brain system that provides temporary storage and manipulation of the information necessary for such complex cognitive tasks,” such as language comprehension, learning, and reasoning [34, 41]. However, since different researchers have defined working memory differently, there exists some variance among which assessment tools actually measure working memory rather than the similar but distinct short-term and long-term memory domains [42]. For example, researchers in one study used the paced auditory serial addition test to measure working memory [43] yet researchers in a separate study used the same test to measure executive function [44]. Therefore, in order to maximize methodological consistency, this review followed an extensive and clearly defined categorization of various cognitive measurements, including working memory and non-working memory tests, used in a previous systematic review [33]. Consequently, studies captured in this review that measured working memory using a test that was categorized under a non-working memory category were accordingly excluded. However, studies that measured working memory using tests not specifically identified in the previous review, under either the working memory category or the other categories, were eligible for inclusion. As a result, the following working memory tests were ultimately included: Digit Span Forward, Digit Span Backward, Letter Digit Span, Tower of London, CANTAB Spatial Working Memory Errors, N-back task (1-back and 2-back), Letter Digit Span, Letter-Number Sequencing, Spatial Running Span task, Verbal Running Span task, and Reading Span task (Daneman Carpenter).

### Search strategy

No protocol was published a priori nor was this review registered with PROSPERO, though the search strategy was pre-defined prior to the search and screen process. Search terms were selected both after examining relevant keywords from prior systematic reviews [30, 33] and after consultation with a literacy librarian specialist yielding the following terms: [physical exercise], [aerobic exercise], [exercise], [aerobic], [“physical activity”], [“resistance training”], [“strength training”], [exertion], [“weight lifting”], [walking], [fitness], [non aerobic physical activity], [non aerobic physical exercise], [“balance training”], [“muscle strength”], [stretching], and [recreation] combined with “or.” Cognitive search terms consisted only of [“working memory”] which was combined with the intervention terms with “and.” Studies were retrieved from Annual Reviews, ProQuest, PubMed, PsycARTICLES, PsycINFO, and Web of Science. The timeline selected was 7 years between August 2009 and December 2016. This range was selected to provide a recent appraisal of studies since the last and most recent review [33] that included PA interventions, healthy individuals as a population of interest, and measured WMP as a cognitive outcome (see Additional file 2).

### Risk of bias assessment

After screening and inclusion of studies were completed, each study was assessed for the presence of various biases using the domain-based evaluation tool devised by Cochrane [45]. Specifically, studies were evaluated as “low risk,” “unclear risk,” or “high risk,” for the presence of selection bias (checking for random sequence generation and allocation concealment), performance bias (checking for blinding of patients and personal), detection bias (checking for blinding of outcome assessment), attrition bias (checking for incomplete outcome data, or the amount, nature, and handling of such data), and reporting bias (checking for selective reporting). For the “other bias” category, this review designated sample sizes as the measurement of interest whereby studies with fewer than 30 participants per treatment arm were identified as “high risk” of bias, studies with equal to or greater than 30 participants per treatment arm were identified as “low risk” of bias, and if sample size data was not reported, then an “unclear risk” of bias would be designated. Finally, all studies were completely assessed by AR with BL’s input for studies with ambiguous or difficult to discern biases.

### Data synthesis and analysis

Studies were extracted and categorized into Zotero, a free and open-source reference management software, and were subsequently screened via Microsoft Excel. Data from included studies were then entered into Review Manager 5.3, including methods, participant characteristics, intervention, and the intensity, volume, and frequency of the respective interventions. Outcome measures were reported as means and standard deviations and inputted accordingly via Review Manager. Additionally, Comprehensive Meta-Analysis Version 3 software was used to conduct moderation analyses on appropriate extracted data as well.

Several meta-analyses were performed via Review Manager that analyzed data from the included studies. All data were continuous in nature, with reported means and standard deviations extracted; in only two cases were standard errors reported that were transformed to standard deviations via Cochrane recommended methodology (see Additional file 3). In addition, partial unpublished data was sought for one study [46]. The majority of studies reported both baseline and final measurement data, but none, other than one study [47], calculated change-from-baseline means and standard deviations. Thus, meta-analyses conducted in this review included only final measurement data. Moreover, Cochrane has explained that meta-analyses using only final measurement data are both an accepted and widely implemented methodology for several reasons albeit with lesser statistical power. First, a common practical problem associated with including change-from-baseline data is that the standard deviations of such data are rarely reported. Second, a common problem of meta-analysts concerns the fact that baseline and final measurement data are drawn from differing numbers of participants due to attrition from either dropout, withdrawal, etc. Such discrepancies create less accurate meta-analysis results, but it is a matter of fact that attrition between baseline and final data characterizes many studies. In regards to RCTs, Cochrane specifically states that using final measurement data will be, on average, the same as using change data, which adds further rationale for extracting final measurement data in this review [38].

Meta-analyses were summarized by Hedges’ adjusted *g* effect sizes, which were interpreted according to Cohen’s scale: trivial, small, moderate, or large [30, 48]. Effect sizes (ES) serve as a quantitative measure of the difference between two groups, in this case between the PA intervention and control groups. Additionally, meta-analyses in this review incorporated a random effects model for several reasons. First, a random effects model accounts for the possibility that other unpublished or overlooked studies were not captured in this systematic review [48]. Second, a random effects model minimizes over-weighing large studies and thereby potentially losing small study effects. Third, a random effects model provides more conservative statistical claims, especially when there is high heterogeneity present [45]. Finally, because the extracted variables are continuous, the differences between fixed and random models are often statically negligible.

Additionally, moderator analyses were performed on various categorical variables, including age via WHO recommended age groups [23], PA intensity, PA duration, presence of allocation concealment, presence of blinding of either participants or researchers, and intervention type (acute or chronic). Analyses were also conducted under a mixed-effects model (random model effects) which stipulates that the given moderator can explain a proportion of variance, but that significant variance can also be explained beyond the captured study data.

## Results

### Study selection

**Fig. 1:**
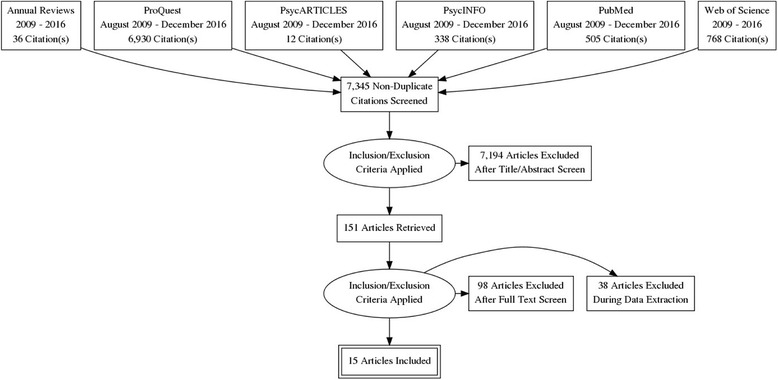
PRISMA flow diagram illustrating databases searched and the subsequent identification, screening, and final inclusion of relevant studies that used RCTs to examine the influence of physical activity on working memory in cognitively and physically healthy individuals

Results from the search strategy were presented according to the recommended PRISMA flow diagram (see Fig. 1). Overall, 8589 studies were reviewed from the six electronic databases with 1244 duplicates identified via Zotero resulting in 7345 unique studies. Next, a three-step screening process was performed on all unique studies. First, studies were first screened by title and abstract to identify articles with RCT design resulting in 554 studies. Second, remaining studies were screened by title and abstract to confirm that the selected RCTs explicitly stated and incorporated PA interventions resulting in 151 studies. Finally, the remaining studies were again screened by title and abstract to confirm that WMP was an outcome being measured, and when necessary, a full-text screen was required if both the title and abstract were ambiguous in outcome measurements (Additional file 4). In total, 15 studies were included for analysis [46, 47, 49–61]. Studies were excluded for several reasons, such as WMP referenced without actually being a measurement outcome (often times in the introduction or discussion); a sample population was impaired physically, cognitively, or both; the PA intervention was dual-task or not physical, i.e., video game; control group contained PA elements, i.e., passive cycling.

### Study characteristics

Cohort characteristics of each study were summarized, including sample characteristics, methodology, eligibility criteria, working memory instrument, and working memory outcome (see Table 1). Overall, the 15 studies yielded a pool of 1315 participants, 686 of which belonged to the PA intervention arms. Per WHO global age guidelines, seven studies had sample population means within the 5–17-year-old group [51, 56–61], three studies had sample population means within the 18–64-year-old group [46, 50, 52], and five studies had sample population means within the 65 years or older group [47, 49, 53–55]. The most common working memory instruments were Digit Span Forward, Digit Span Backward, and N-back tasks followed by several miscellaneous instruments. Further, nine studies included more than one instrument measuring WMP. Also of note, of the 13 working memory instruments included, five instruments were not in the previously defined working memory categorization [33]. With regards to attrition, only two studies [47, 53] did not meet the recommended attrition threshold according to Cochrane guidelines [45]. Lastly, blinding of either participants or personnel/researchers was only present in four studies [51, 53–55].

**Table 1:** Cohort characteristics of included studies

Citation	Sample characteristics	Study characteristics	Inclusion/Exclusion criteria	Instrument(s)	Did working memory improve?
Brown, 2009	*N* = 154 Age: 62 to 95 M = 79.6 % Female: 88	Allocation: cluster randomization Blinding: No Attrition: 18%	Inclusion: NR Exclusion: neurological, cardiovascular or musculoskeletal problem that precluded safe exercise participation, <20 MMSE, already attending exercise classes	WAIS-R Digit Span Forward and Backward	No
Budde, 2010	*N* = 60 Age: 15 to 16 M = 14.37 % Female: 43	Allocation: randomization Blinding: NR Attrition: 2%	Inclusion: absence of dyslexia, a maximum BMI of 25, absence of mental or physical impairments, and no history of psychoactive substances Exclusion: NR	Letter Digit Span	Yes, but only for low performers
Chang, 2011	*N* = 42 Age: NR M = 21.97 % Female: 69	Allocation: mixed randomization Blinding: NR Attrition: 0%	Inclusion: healthy history questionnaire, IPAQ, PAR-Q “no” for all responses, assessments to ensure no potential risk factors to impair aerobic exercise Exclusion: NR	Tower of London	Yes
Fisher, 2011	*N* = 64 Age: NR M = 6.1 % Female: 55	Allocation: randomization Blinding: Yes Attrition: 3%	Inclusion: no diagnosed cognitive disorder, physical impairments Exclusion: reaction time to ANT is <200 ms indicating anticipatory responding	CANTAB Spatial Working Memory Errors	Yes
Hariprasad, 2013	*N* = 120 Age: ≥60 Intervention: M = 75.74 Control: M = 74.78 % Female: 83	Allocation: block randomization Blinding: Yes Attrition: 28%	Inclusion: >60 year olds from consenting elderly homes Exclusion: “using MINI to exclude dementia or other neurodegenerative disorders, stroke, major depressive disorder, psychosis, anxiety disorder, severe hearing and visual impairment and inability to perform yoga practices; evaluated by clinician to rule out depression, dementia, and other psychiatric disorders; scores of >4 GDS, <26 HMSE excluded”	WMS Digit Span and WMS Spatial Span	Yes
Hogan, 2013	*N* = 144 Age: 19 to 93 Intervention: M = 51.34 Control: M = 50.79 % Female: 49	Allocation: stratified randomization Blinding: NR Attrition: 0%	Inclusion: negative response for all PAR-Q, score of ≥23 MMSE, or received verbal permission from his or her doctor for participation Exclusion: NR	N-back task (2-back)	Yes, faster reaction times reported
Chen, 2014	*N* = 87 Age: 3rd and 5th grade 3^rd^ grade: 9.18 5^th^ grade: 11.11 % Female: 48	Allocation: stratification randomization Blinding: No Attrition: 0%	Inclusion: “scores of PAR-Q = 6, >90 WISC-IV-C, >160 SCL-90-C, no attention deficit/hyperactivity disorder, right handed, normal or corrected-to-normal vision, not color-blinded, and not be taking psychoactive medications” Exclusion: NR	N-back task (2-back) modified	Yes, but only for 5^th^ grade group
Gothe, 2014	*N* = 118 Age: 55 to 79 M = 62.0 % Female: 83	Allocation: randomization Blinding: NR Attrition: 8%	Inclusion: “participants had to be between 55 and 79 years of age, scores of >21 TICS, ≥5 GDS required, English speaking, report being sedentary for at least the previous 6 months, have no on-going regular yoga practice, be able to get up and down from the floor, have good or corrected vision (20/40), and be willing to be randomized into one of two exercise groups” Exclusion: NR	N-back task (1-back and 2-back)	Yes
Nouchi, 2014	*N* = 64 Age: 60 and older Intervention: M = 75.74 Control: M = 67.06 % Female: NR	Allocation: randomization Blinding: Yes Attrition: 5%	Inclusion: “right-handed, native Japanese speakers, unconcerned about their own memory functions, not using medications known to interfere with cognitive functions, and having no disease known to affect the central nervous system; did not exercise regularly, not members of a gym/health club, and not participating in another exercise study” Exclusion: scores of <85 JART, <26 MMSE, 12 < FAB, participation in other cognitive-related studies	WAIS Digit Span Forward and Backward	No
Vaughn, 2014	*N* = 49 Age: 65 to 75 Intervention: M = 69.0 Control: M = 68.8 % Female: 100	Allocation: randomization Blinding: Yes Attrition: 2%	Inclusion: screening via age, gender, amount of weekly exercise, ability to walk 20 m, availability, TICS, PAR-Q Exclusion: cognitive impairment via ≥31 TICS, dementia, Parkinson’s disease, or recent head injury	Letter-Number Sequencing	Yes
Bantoft, 2015	*N* = 45 Age: NR M = 22.67 % Female: 71	Allocation: counterbalanced randomization Blinding: NR Attrition: 0%	Inclusion: screening via WTAR, HADS Exclusion: pregnancy, heath conditions (i.e. heart disease, chronic back pain)	Digit Span Forward and Backward WAIS Letter-Number Sequencing	No
Basso, 2015	*N* = 92 Age: 18 to 35 M = 22.21 % Female: 60	Allocation: randomization Blinding: NR Attrition: 8%	Inclusion: NR Exclusion: had major surgery within prior 6 months, past or present history of drug or alcohol abuse, had a diagnosed psychiatric or neurological condition, taking medication known to affect cognition, unable to safely participate in an aerobic exercise program	Digit Span	Yes
Howie, 2015	*N* = 96 Age: 9 to 12 M = 10.7 % Female: 65	Allocation: randomization Blinding: NR Attrition: 2	Inclusion: NR Exclusion: NR	Digit Span modified	No
Albinet, 2016	*N* = 41 Age: 60 to 80 Intervention: M = 67 Control: M = 66 % Female: 72	Allocation: randomization Blinding: NR Attrition: 12%	Inclusion: screened by personal physician “who rated them as being in good health”, retired, aged between 60 and 80 years, ≥26 MMSE, physically sedentary via DSPA, able to swim, agreement to be randomized Exclusion: taking medication that could affect cardiovascular health or cognitive functions, carrying a pacemaker, cardiorespiratory or neurological disease, major surgery within one year prior to testing	N-back task (2-back) Spatial Running Span task Verbal Running Span task	Yes
Chapman, 2016	*N* = 67 Age: 56 to 75 Intervention M = 63.5 Control M = 64 % Female: 73	Allocation: block randomization Blinding: NR Attrition: 18%	Inclusion: no history of neurological or psychiatric conditions, normal IQ range, native English speakers, and minimum of high school diploma Exclusion: MR scanning contraindications, cognitive status (TICS-M <28 and MoCA <26), depression indication (BDI-II >14), left-handedness, body mass (BMI >40, kg/m^2^)	Reading Span Task (Daneman Carpenter)	No

*ANT* Attention Network Test, *BDI* Beck Depression Inventory, *BMI* Body Maximum Index, *CANTAB* Cambridge Neuropsychological Test Battery, *DSPA* Dijon Score of Physical Activity, *FAB* Frontal Assessment Battery, *GDS* Geriatric Depression Scale, *HADS* Hospital Anxiety and Depression Scale, *HMSE* Hindi Mental Scale Examination, *IPAQ* International Physical Activity Questionnaire, *JART* Japanese Reading Test, *MINI* Mini International Neuropsychiatric Interview, *MMSE* Mini Mental State Exam, *MoCA* Montreal Cognitive Assessment, *PAR-Q* Physical Activity Readiness-Questionnaire, *SCL* Symptom Checklist-90-Chinese Version, *TICS* Telephone Interview for Cognitive Status, *WAIS-R* Wechsler Adult Intelligence Scale-revised, *WISC-IV-C* Wechsler Intelligence Scale for Children-IV-Chinese, *WMS* Wechsler Memory Scale, *WTAR* Wechsler Test for Adult Reading, *NR* not reported

Characteristics of the PA interventions were summarized, including intensity, volume, frequency, setting, format, and control condition (see Table 2). The majority of studies were characterized by a single PA intervention with several studies integrating multimodal interventions [47, 54, 55]. Present in seven studies, aerobic PA was the most frequently utilized intervention type; control conditions varied more with waitlist as the most frequent designated control. There was noticeable variability with intensities ranging from “light to moderate” to “vigorous” with four studies, two of which were yoga-based modalities, not reporting intensity of their respective interventions [47, 53–55]. Studies that reported their intensities in maximum heart rate or maximal oxygen uptake [50, 54] were converted to their categorical analogue via American College of Sports Medicine guidelines [62]. As for other characteristics, the volume of interventions in 11 studies lasted 30 min or longer per session with ten studies utilizing the group format with all individual formats conducted under a laboratory setting. Finally, and most importantly, eight studies were identified as chronic PA studies [47, 49–55] defined by having more than one PA session (duration ranging from 4 weeks to 6 months) with the remaining seven studies identified as acute PA studies [46, 56–61] defined by having only one PA session.

**Table 2:** Intervention characteristics included studies

Citation	Intervention modality	Intensity	Volume (min)	Frequency	Duration	Format	Setting	Control condition
Brown, 2009	Resistance, balance training, motor fitness	NR	60	2 days/week	6 months	Group	Retirement Village	No-exercise
Budde, 2010	Running	Moderate	12	1 session	1 session	Group	400-m track	Sedentary
		Vigorous						
Chang, 2011	Aerobic exercise via cycle ergometer	Moderate to vigorous	30	1 session	1 session	Individual	Laboratory	Health education
Fisher, 2011	Aerobic exercise	Moderate to vigorous	120	2 days/week	10 weeks	Group	Primary school	Skill development
Hariprasad, 2013	Yoga	NR	60	Daily in Month 1, weekly in Month 2 and 3	6 months	Group	Old-age home	Waitlist
Hogan, 2013	Stationary cycling	Moderate	15	1 session	1 session	Individual	Laboratory	Health education
Chen, 2014	Jogging	Moderate	30	1 session	1 session	Group	School	Health education
Gothe, 2014	Hatha yoga	NR	60	3 days/week	8 weeks	Group	Community center	Stretching-strengthening
Nouchi, 2014	Aerobic, strength, stretching	Moderate to vigorous	48	3 days/week	4 weeks	Group	Sendai city, Miyagi prefecture, Japan	Waitlist
Vaughn, 2014	Aerobic, strength, stretching	NR	60	2 days/week	16 weeks	Group	Community center	Waitlist
Bantoft, 2015	Walking via treadmill	Low	≤60	1 session	1 session	Individual	Laboratory	Sit workstation
Basso, 2015	Aerobic exercise via stationary bicycle	Vigorous	50	1 session	1 session	Individual	Laboratory	Video watching group
Howie, 2015	Aerobic exercise via Brain BITES	Moderate to vigorous	10	1 session	1 session	Group	School	Sedentary classroom activity
Albinet, 2016	Aquaerobics and swimming	Moderate to vigorous	60	2 days/week	5 months	Group	Senior community center	Stretching-flexibility exercises
Chapman, 2016	Aerobic exercise	Moderate to vigorous	60	3 days/week	12 weeks	Individual	Laboratory	Waitlist

*Brain BITES* Better Ideas Through ExerciSe, *NR* not reported

**Fig. 2:**
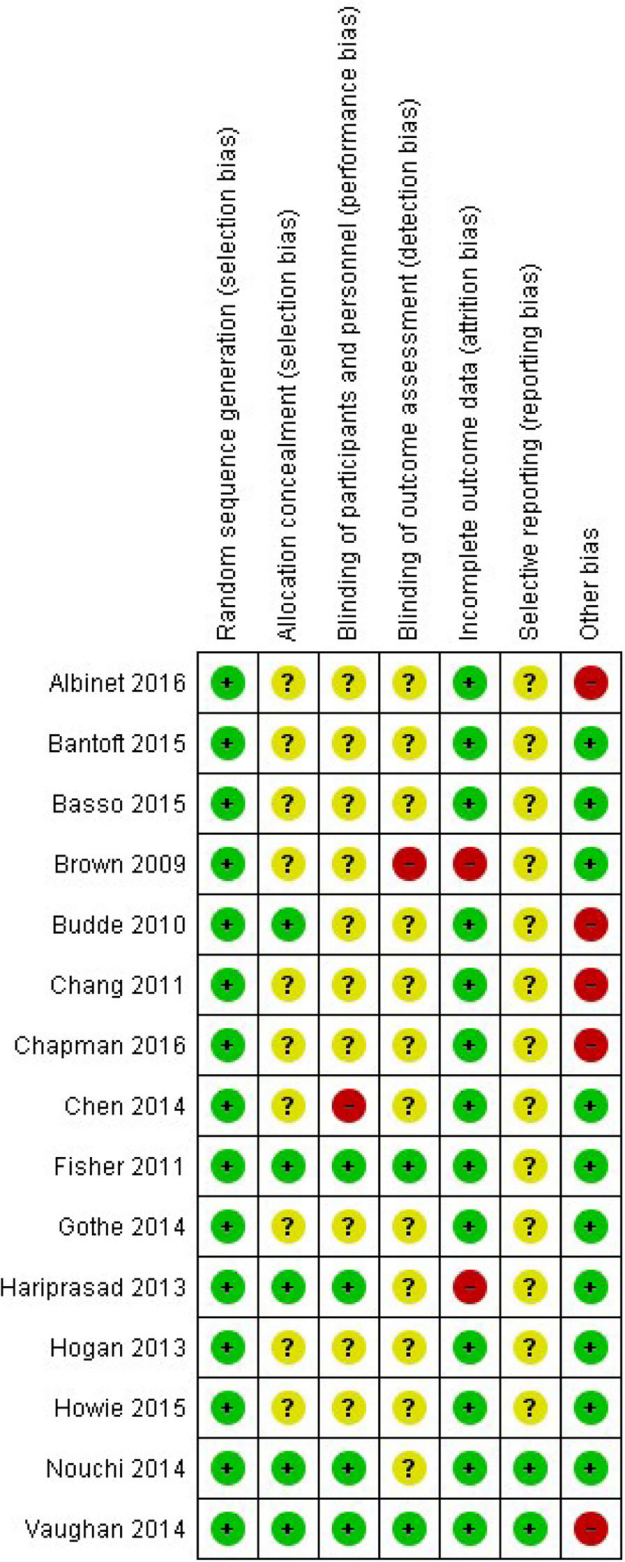
Risk of bias summary: review authors’ judgements about each risk of bias item for each included study

Taken together, all 15 studies were individually assessed via a risk of bias summary chart (see Fig. 2). Overall, a low risk of both selection and attrition bias characterized the vast majority of the studies with the exception of two chronic PA studies [47, 53] having a high risk of attrition bias. Conversely, the “other bias” category (sample size) was the most common bias to be of high risk with five total studies [49, 50, 55, 58, 59], three of which were chronic PA studies. The chart also reveals that, of the 15 studies, the top four with the least amount of cumulative bias present were chronic PA studies, although no study was completely characterized by low risk of bias. With regard to blinding, only two studies [51, 55] were characterized with low risk for both performance and detection bias. Similarly, only two studies were characterized with low risk of reporting bias [54, 55] having been the only studies with a priori protocols. Indeed, the risk of bias assessment offers interesting commentary on current literature quality that will be discussed in the forthcoming section.

### Synthesis of results

Fifteen studies were included [46, 47, 49–61], ten (67%) reporting that PA conferred a statistically significant improvement in WMP in at least one measured outcome with the remaining five studies finding no significant improvement. When stratified by intervention, five of the eight chronic PA studies (>1 session) and five of the seven acute PA studies (1 session) showed significant improvements in WMP. In addition to the descriptive analysis of each study, meta-analyses were conducted for quantitative analysis. Prior to these empirical tests, two studies were split [58, 59] into two additional units of analyses because each used distinct, stratified populations with specific intervention and control conditions that would make averaging them together erroneous; effectively, 17 unique studies were analyzed. As well, due to nine studies measuring WMP via multiple instruments, a total of 28 outcomes were ultimately included and examined for meta-analysis.

**Fig. 3:**
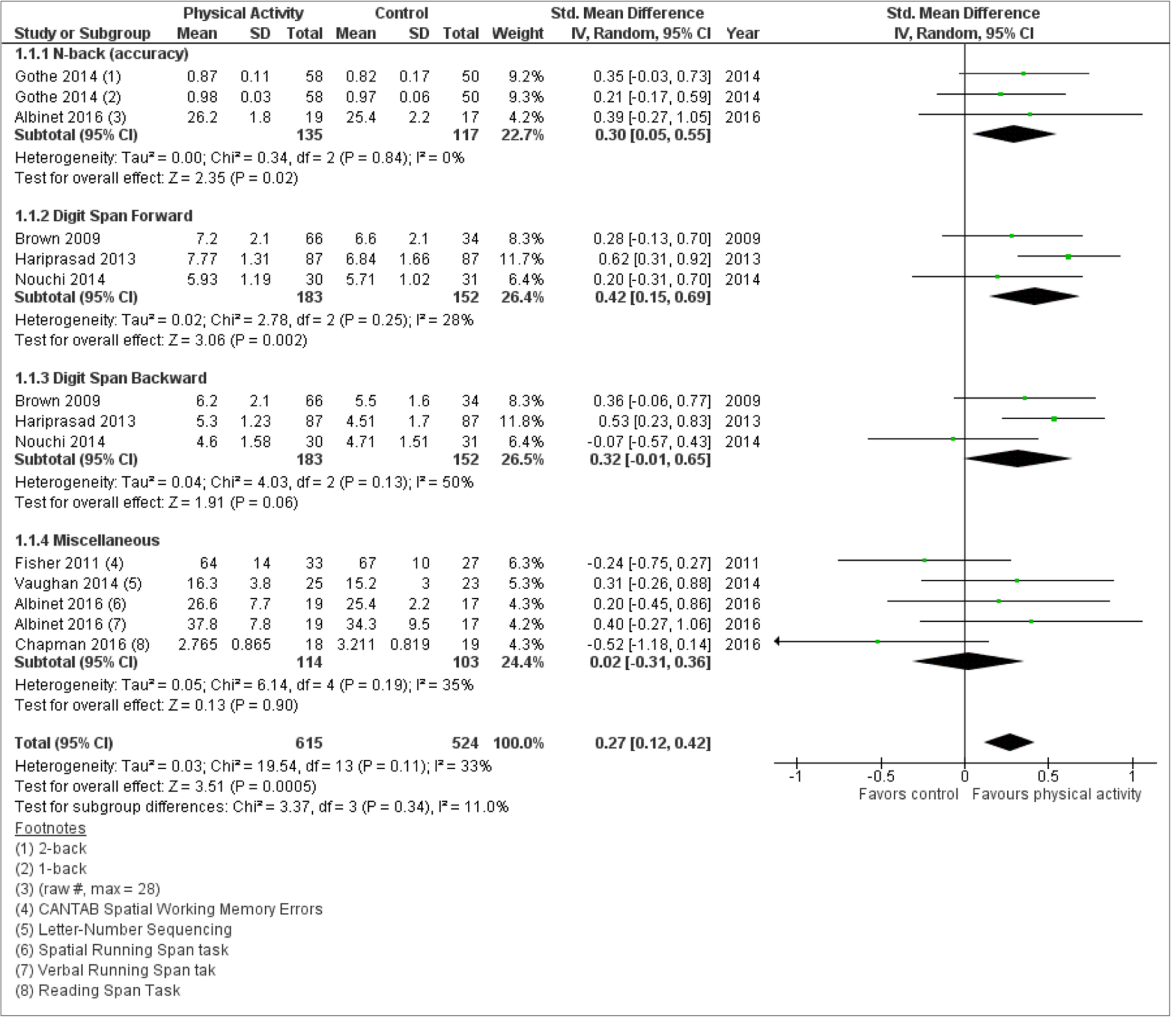
Eight included studies that measured chronic physical activity were organized by the type of working memory test. The figure provides subgroup meta-analyses, as well as a cumulative meta-analysis. The calculated difference between physical activity and control groups are considered statistically significant given the total diamond does not cross the “line of no effect”

**Fig. 4:**
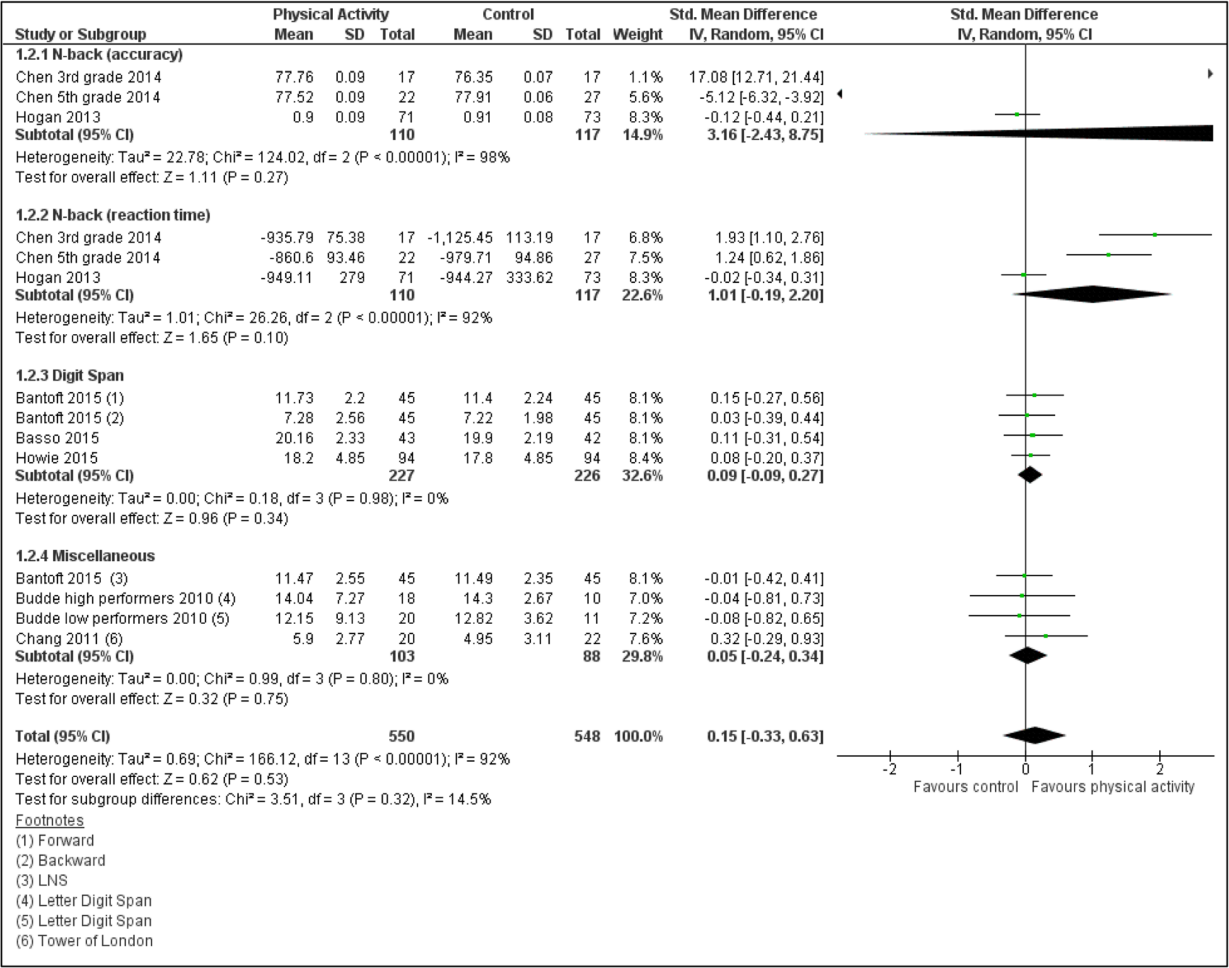
Acute PA on WMP Meta-analysis. Seven included studies that measured acute physical activity were organized by the type of working memory test. The figure provides subgroup meta-analyses, as well as a cumulative meta-analysis. The calculated difference between physical activity and control groups is considered not statistically significant given the total diamond crosses the “line of no effect”

Two separate meta-analyses were performed, one examining chronic PA studies [47, 49–55] and the other examining acute PA studies [46, 56–61] with each analyzing 14 outcomes respectively. Analysis of chronic PA studies yielded a significant (*p* = 0.0005) small effect size (ES: 0.27; 95% CI: 0.12, 0.42) that was characterized by non-significant heterogeneity (*I* ^2^ = 33%) with 615 total intervention group participants and 524 total control group participants (see Fig. 3). In contrast, analysis of acute PA studies yielded a non-significant (*p* = 0.53) small effect size (ES: −0.15; 95% CI: −0.33, 0.63) characterized by significant heterogeneity (*I* ^2^ = 92%) with 550 total intervention group participants and 548 total control group participants (see Fig. 4). Taken together, chronic PA had a significant, positive, and small effect on WMP with no evidence that the eight included studies were significantly dissimilar whereas acute PA had a non-significant, positive, and trivial effect on WMP with strong evidence that the seven included studies were significantly dissimilar.

### Moderation analyses

Moderation analyses were performed on categorical variables comparing all studies together irrespective of whether the intervention was acute or chronic (see Additional file 5). For studies with multiple outcomes, their effect sizes were pooled resulting in 17 units of analysis. Categorical variables included WHO recommended age groups (5–17, 18–64, and 65 years or older), PA intensity, presence of allocation concealment, presence of blinding of either participants or researchers, and intervention type (acute or chronic PA). Null hypothesis for each categorical variable was that no moderator effect exists between PA and WMP.

Moderation analysis of age revealed the variable to be a significant moderator (*Q* = 11.202, *p* = 0.004) when comparing studies with sample mean ages of 5–17 years (*n* = 9), 18–64 years (*n* = 3), and 65≥ years (*n* = 5). The test of null was significant (*p* < <0.05) only for studies with average participant ages of 65≥ yielding a positive and small effect size (ES: 0.324; 95% CI: 0.185, 0.463). In other words, those five studies [47, 49, 53–55], all which were chronic PA based, the effect size was not zero. Overall, analysis of the data indicates that age is a positive moderator between PA and WMP, i.e., as age increases so does improvement in WMP and significantly so for those 65 years or older specifically.

Prior to analysis of PA intensity, several data ransformations were required to achieve three groups (low, moderate, moderate to vigorous). Of note, four studies did not report intensities for their interventions [47, 52, 53, 55], and after reviewing each intervention more closely, two of which were yoga centered [52, 53], all four were subsequently reported as “low” intensity. Additionally, only one study indicated a “vigorous” PA intensity [57], which was changed to join the eight studies with “moderate to vigorous” PA intensity. We found intensity was a significant moderator (*Q* = 7.399, *p* = 0.025) between low (*n* = 5), moderate (*n* = 3), and moderate to vigorous (*n* = 9) PA intensities. The test of null for studies with low PA intensity (four of which were chronic PA) was the only one to be significant (*p* = 0.001) with a small effect size (ES: 0.269; 95% CI: 0.109, 0.430). However, we caution inferences about this analysis due to the transformations of the data variable (for instance, if the single study with “vigorous” intensity was included then the analysis would have been non-significant). In sum, though, our data suggest that intensity acts as a moderator variable between PA and WMP. Next, analysis of allocation concealment was revealed as a non-significant moderator (*Q* = 0.114, *p* = 0.736) between studies that did conceal allocation (*n* = 6) and studies that did not state nor describe any allocation concealment (*n* = 11). Further, neither category of allocation yielded a significant test of null. Similarly, blinding was also revealed to be a non-significant moderator (*Q* = 0.401, *p* = 0.818) between studies that included blinding (*n* = 4), did not include blinding (*n* = 3), or did not describe any blinding in their design (*n* = 10); no significant test of null results were identified either. Ultimately, neither allocation concealment nor the presence/absence of blinding were shown to be moderators between PA and WMP.

Finally, the intervention type (chronic or acute PA) was also found not to be a significant moderator (*Q* = 2.384, *p* = 0.123). The test of null, however, was significant for chronic PA studies (*p* = 0.040) for a small effect size (ES: 0.190; 95% CI: 0.009, 0.370), which indicates that the true effect size was not zero for this group of studies. In other words, improvements in WMP did occur when the intervention was chronic; however, there lacked total sufficient evidence from all studies to claim that intervention type could be a moderator between PA and WMP.

## Discussion

This systematic review included 15 RCT studies that investigated a physical activity intervention on WMP among healthy sample populations with eight chronic PA-centered studies and seven acute PA-centered studies. Analysis of the studies included summarized cohort and intervention characteristics, risk of bias assessment, meta-analyses, and moderator analyses. While previous reviews focused almost exclusively on cognitively impaired populations, measured multiple cognitive outcomes, or contained broad study design inclusions, this review was designed and completed with a focused, novel research question that has not yet been purposefully investigated.

Overall, this review found that PA interventions from ten studies conferred a significant improvement in WMP. Specifically, five of eight chronic PA studies and five of seven acute PA studies were associated with WMP improvements. Meta-analyses revealed a significant albeit small, positive outcome that healthy participants who engaged in chronic PA interventions yielded significant improvements in their WMP, which stands in contrast with previous literature [33] that had found chronic PA conferring no WMP improvement. Moreover, the fact that our analysis was characterized by non-significant heterogeneity indicates that the eight studies were not significantly dissimilar from each other adding further confidence to the result. With regard to the ES, one likely obstacle towards yielding a larger statistic was related to the diversity of working memory instruments included in our review, specifically under the “Miscellaneous” subgroup. Although our review relied on a previous categorization of working memory instruments [33], there remains some debate on how well certain instruments actually measure working memory relative, to say, attention control or other domains [63]. In fact, when the Miscellaneous subgroup was removed, the ES increased from small to moderate (ES: 0.37, *p* < <0.001). Thus, while it is desirable to have as many included studies utilizing the same WMP tests to help increase the likelihood of significant meta-analyses, and with a stringent exclusion criterion such goals are possible, such reviews may suffer from limiting its generalizability. In contrast, meta-analysis of the seven acute PA studies revealed a non-significant, positive, and trivial ES with evidence that the studies of this cohort were significantly dissimilar. Given this result, only factors that may have prevented a significant result can be discussed. For instance, compared to the chronic PA analysis, there was greater study weight disparity along with substantial heterogeneity between the seven acute PA studies. As for this analysis, while removal of the Miscellaneous subgroup led to the ES increasing from trivial to small, the change remained non-significant suggesting the the limiting factors were beyond the selection of WMP instruments. For instance, none of the seven acute PA studies included samples in the 65≥ mean age group which, as our moderation analysis showed, was a significant interaction variable for the five chronic PA studies with 65≥ age group. Thus, it is possible that age also played a limiting factor towards achieving significant results. From a conceptual view, acute PA interventions may not lead to immediate effects in WMP improvements from a single PA session, as evidenced by the studies in this review [56, 61]; however, there are examples elsewhere in the literature that show significant cognitive benefits from acute PA interventions [64, 65]. As for chronic PA interventions, prior research [1, 33] has suggested that interventions ranging from 4 to 12 weeks can offer robust cognitive improvements, and indeed, all eight chronic PA studies captured in this review were within this range. Thus, chronic interventions allow for improvements in WMP to accumulate throughout study duration so that by the study’s conclusion the effect has built up yielding larger post-intervention scores.

The risk of bias assessment showed that acute PA studies suffered more instances of unclear and high risk of biases, particularly with respect to allocation concealment and blinding. Intuitively, chronic and acute PA studies are designed with different parameters and considerations in mind. For instance, with respect to blinding, acute PA studies may not have incorporated blinding in their study design either due to funding or because it may have been impractical, i.e., the researchers were the same personnel observing the participants in the laboratory (four of the seven acute PA studies were laboratory based). In contrast, blinding was more common among chronic PA study designs, including blinding of participants [53], blinding of personnel, such as group instructors for a given PA regimen [54], or blinding of researchers themselves upon analyzing data [51]. It is likely that prospective studies that last approximately weeks to months must arguably account for more variables and biases given the nature of their studies, and are more likely to have more funding as a result. Regardless of funding or impracticality, the fact remains that acute PA studies where characterized by more uncertainty of bias regarding performance and detection bias. As for the risk of bias assessment itself, this review chose conservative designations of biases. For instance, if a study did not mention any blinding, it was designated as an unclear risk of bias. However, one could argue that for a study not mentioning the presence of blinding could be a tacit admission that blinding was *not* incorporated in their study. Although possible, Cochrane guidelines suggest that if no mention of the bias is addressed, there is then insufficient evidence to make a claim as to high or low risk of bias. Indeed, it was the fact that allocation concealment and blinding were shown to be less common among acute PA studies that served partially as the impetus for measuring both variables among others during moderation analyses.

Moderation analyses helped identify certain categorical variables such as age and PA intensity as significant while others such as allocation concealment, blinding, and intervention length as non-significant. However, moderation analyses are strictly observational in nature—the ability to make causal inferences is not appropriate nor permissible, rather only observable statements can be stated. As well, the more significant test of null results present for individual groups in a given analysis the stronger the case for the observed moderator. Age was identified as a significant moderator when comparing age groups of 5–17, 18–64, and 65≥ years. That is, age interacts and moderates between the PA intervention and the WMP outcome. Specifically, it was observed that as age increases so does the ESs, although between the three age groups only the 65≥ years had a significant test of null. In other words, it was possible that the 5–17 and 18–64 age groups had ESs that could have been zero, while 65≥ years did not. As previously addressed, none of the acute PA studies had mean age of 65≥ years which likely limited the extent of WMP improvements. Intensity was also a significant moderator between PA intervention and WMP performance with only the low intensity PA interventions yielding a significant test of null result, although inferences about this observation are cautioned given that nearly 30% of the data was transformed prior to analysis. Next, both allocation concealment and blinding were not significant moderators with no significant test of null results. Finally, the intervention type (chronic or acute PA) was also tested for a potential moderator variable. As stated in the “Background” section, our underlying assumption, for which the literature strongly supports, is that chronic and acute PA interventions operate under different mechanisms with potential to yield different cognitive effects. Thus, we choose to review and analyze data according to this paradigm and so examining intervention type under the moderation analysis would serve as an affirmation that our data followed the stated paradigm. Therefore, for the intervention to be identified as a non-significant moderator was a revealing result suggesting that the 15 included studies may not be large enough to detect the differential effects between chronic and acute PA studies. However, a significant test of null result for chronic PA studies indicated that acute PA studies likely were the source for the overall non-significant result. As previously stated, these differences likely point to differences in study design and parameters, as well as the overall quality of the individual studies since it is known that acute PA can yield cognitive improvements. Overall, while only two of the five moderators analyzed yielded significant results these results can help identify categorical variables that can guide future research directions.

### Limitations

There were several limitations present in this systematic review. First, since this systematic review followed a strict and narrow research question, it limits itself in generalizing knowledge to other cognitive domains, such as executive function and attention. Second, although using the preferred term of “physical activity” may have increased the number of potential studies to be included, the term “physical exercise” provides a more concrete term that confers a more structured regimen of activity. That is, if this review only searched for physical exercise interventions then the variance between intervention characteristics would likely have decreased, and perhaps, more significant results would have been produced. Specifically, some could argue that the inclusion of yoga-based interventions and walking extend the definition of PA beyond more conventional forms, such as cycling or resistance training. However, with yoga’s increasing popularity in the Western world, and with an aging population, alternative forms of PA as well as the light PA activity of walking are currently under investigation for their possible protective effects [66]. Fourth, the objective of this review focused on searching recent publications within the previous 7 years with the intention of acting as an update since the most recent review that reported on a similar topic [33]. However, some previous reviews investigating PA and cognition developed their search ranges approximately by decades [1, 30, 33], and so relative to our search range, it is possible to view it as a restrictive and conservative date range. Because studies published before August 2009 were excluded, there is a strong likelihood that there were articles that not only satisfied our inclusion criteria, but could also have added significant data to alter the complexion of our meta-analyses with further power and generalizability. Fifth, there were very few within-subject design RCTs [56, 61] which are a more rigorous study design as it limits individual variability in cognitive performance. Lastly, PRISMA guidelines recommend protocols be published a priori, and the authors of this review did not do so as the decision to use both descriptive and analytical tools was decided after the completion of the search strategy, among several other analytical decisions determined once our data was already collected.

## Conclusion

This review found statistically significant evidence that chronic physical activity conferred improvement in WMP in the selected studies of healthy subjects while acute physical activity studies did not confer any significant result in WMP. With respect to chronic PA, this outcome aligns with previous literature [15, 17]; however, this review is one of the few reviews that exclusively investigated WMP and found a positive effect as well. Although conventional thinking suggests that PA should improve, even minimally, cognitive function, this conclusion has not always been supported when systematic reviews compile and integrate data from individual studies [28, 29]. Indeed, the contrasting results between chronic and acute PA studies offers interesting future directions to explore different mechanisms that govern both intervention types. However, both chronic and acute PA studies suffered from similar deficits. For one, the risk of bias assessment revealed a much larger proportion of “unclear risk” of bias for many relevant categories, including blinding, allocation concealment, and selection bias. While not unique to PA and cognition studies [67], these biases are strongly encouraged to be addressed in future studies. In fact, and perhaps counterintuitively, studies with “high risk” of bias had at least identified the presence of such biases and accordingly were more transparent thereby adding to the quality of their study in this regard [47, 60]. In contrast, studies with an “unclear risk” of bias provided insufficient evidence to make a determination and can leave readers unsure of whether it was not addressed due to impracticality, fear of publication bias, or other reasons. To that end, the authors of this review strongly encourage future studies to explicitly state the presence or absence of the biases that the Cochrane risk of bias assessment tool has identified—doing so not only improves transparency but also helps in the overall evaluation of such studies.

Despite the overall mixed results from this systematic review, additional considerations provide credibility and potential value for other interested researchers. First, whereas previous reviews have focused on multiple cognitive outcomes with varied sample populations, to our knowledge, this review is the first attempt at exploring the effects of physical activity exclusively on WMP in healthy participants solely through RCTs. Furthermore, according to the Oxford Centre for Evidence-Based Medicine, this systematic review is considered level 1-a evidence, the highest possible designation, which should demonstrate that despite some null results in this review the quality of such results are fairly strong and credible, particularly because only RCTs were included [68]. Further, the underlying goal of pursuing this research question was that this newly synthesized data, both descriptive and analytic, would benefit future researchers in understanding the association between PA and WMP, and hopefully add further evidence towards why PA is a recommended, low-cost activity for improving one’s cognition. In other words, knowing how much of an impact PA can have on the cognitive function of healthy people can further emphasize the importance of studies that show improvement in the cognitively impaired [5–12]. Based on the overall results, we recommend and encourage future researchers to test chronic, low intensity PA interventions in elderly populations of 65≥ in order to increase the likelihood of significant results regarding WMP. Alternatively, we also recommend that acute PA interventions continue to be explored considering the lack of significant results from this review. Moreover, researchers should strive to conduct RCT-based trials that adhere to recommended guidelines listed by international and credible organizations [69]. Certainly, with increased scientific interest in the domain of PA and cognition, more systematic reviews should be pursued and published to provide timely and worthy insights on the differences and similarities between chronic and acute PA and their respective effects on WMP in healthy individuals.

## Additional files

Additional file 1: PRISMA Checklist. (DOCX 25 kb)

Additional file 2: Search strategy. (PDF 20 kb)

Additional file 3: Extracted data. (PDF 110 kb)

Additional file 4: Raw screening data. (XLSX 6436 kb)

Additional file 5: Moderation analyses. (PDF 226 kb)

## Abbreviations

CI: Confidence interval
ES: Effect size
PA: Physical activity
PICOS: Population Intervention Comparator Outcome Study design
PRISMA: Preferred Reporting Items for Systematic Review and Meta-Analysis
RCT: Randomized controlled trial
WMP: Working memory performance
