# Supplementary material for: The effects of chronic and acute physical activity on working memory performance in healthy participants: a systematic review with meta-analysis of randomized controlled trials
Source: Syst Rev. 2017 Jun 30;6:124. doi: 10.1186/s13643-017-0514-7 (PMC5493123; doi:10.1186/s13643-017-0514-7)
Supplement: Supplementary file 2 — Search strategy. (PDF 20 kb) [file 13643_2017_514_MOESM2_ESM.pdf]

Search Strategy

- PubMed
    - Search terms inserted using “Advanced Search” yielding the following syntax: ((((((((((((((((physical exercise) OR aerobic exercise) OR exercise) OR aerobic) OR "physical activity") OR "resistance training") OR "strength training") OR exertion) OR "weight lifting") OR walking) OR fitness) OR non aerobic physical activity) OR non aerobic physical exercise) OR "balance training") OR "muscle strength") OR stretching) OR recreation) AND "working memory"
    - Filter: “Publication dates” (08-01-2009 to 12-31-2016)
    - 505 results
- ProQuest
    - Databases searched included:
        - Biological Science Database
        - ComDisDome
        - ebrary® e-books
        - Nursing & Allied Health Database
        - ProQuest Central
    - Advanced Search was not used due to more search terms than allowed to insert, and thus the syntax created in the PubMed search was pasted directly into the Search function
    - Filters: “Peer reviewed”, “Publication date” (08-01-2009 to 12-31-2016), “Scholarly Journals”, and “English”
    - 6,930 results
- PsycINFO via EBSCOhost
    - Advanced Search used
    - Filters: “Boolean/Phrase”, “Peer Reviewed”, “Exclude Dissertation”, “Publication Date” (08-2009 to 12-2016)
    - 338 results
- PsycARTICLES via EBSCOhost
    - Advanced Search used
    - Filters: “Boolean/Phrase”, “Scholarly (Peer Reviewed) Journals”, “Exclude Book Reviews”, “Exclude Non-Article Content”, “Publication Date” (08-2009 to 12-2016)
    - 12 results
- Annual Reviews
    - Advanced Search was not used due to more search terms than allowed to insert, and thus the syntax created in the PubMed search was pasted directly into the Search function
    - Filters: “Publication Date” (2009 to 2016)
    - 36 results
- Web of Science (All databases)
    - Advanced Search used
    - Filters: “Publication Date” (2009 to 2016), “Article”, “English”
    - 768 results
