# Supplementary material for: The effects of chronic and acute physical activity on working memory performance in healthy participants: a systematic review with meta-analysis of randomized controlled trials
Source: Syst Rev. 2017 Jun 30;6:124. doi: 10.1186/s13643-017-0514-7 (PMC5493123; doi:10.1186/s13643-017-0514-7)
Supplement: Supplementary file 3 — Extracted data. (PDF 110 kb) [file 13643_2017_514_MOESM3_ESM.pdf]

Citation	Physical Activity Mean, SD	Total (N)	Control Mean, SD	Total (N)	Working Memory Instrument(s)
Brown, 2009	DS-F post: 7.2 (2.1)<br>DS-B post: 6.2 (2.1)	66	NEC DS-F post: 6.6 (2.1)<br>NEC DS-B post: 5.5 (1.6)	34	WAIS-R Digit Span Forward WAIS-R Digit Span Backward
Budde, 2009[1]	LDS LP post: 12.15 (9.13)	LP: 20	LDS LP post: 12.82 (3.62)	LP: 11	Letter Digit Span
	LDS HP post: 14.04 (7.27)	HP: 18	LDS HP post: 14.30 (2.67)	HP: 10	
Chang, 2011	Correct score post: 5.90 (2.77)	20	Correct score post: 4.95 (3.11)	22	Tower of London
Fisher, 2011	Spatial working memory errors post: 64 (14)	33	Spatial working memory errors post: 67 (10)	27	CANTAB Spatial Working Memory Errors
Hariprasad, 2013	DS-F post: 7.77 (1.31)<br>DS-B post: 5.30 (1.23)	87	DS-F post: 6.84 (1.66)<br>DS-B post: 4.51 (1.70)	87	Digit Span Backward<br>Digit Span Forward
Hogan, 2013	RT post (ms): 949.11 (279)<br>AC post: .90 (0.09)	71	RT post (ms): 944.27 (333.62)<br>AC post: .91 (0.08)	73	N-back task (2-back)
Chen, 2014	RT post: 935.79 (75.38)<br>AC % post: 77.76 (0.09)	3rd grade: 17	RT post: 1125.45 (113.19)<br>AC % post: 76.35 (0.07)	3rd grade: 17	N-back task (2-back) modified
	RT post: 860.60 (93.46)<br>AC % post: 77.52 (0.09)	5th grade: 22	RT post: 979.71 (94.86)<br>AC % post: 77.91 (0.06)	5th grade: 27	
Gothe, 2014	AC % 1-back post: 0.98 (0.03)<br>AC % 2-back post: 0.87 (0.11)	58	AC % 1-back post: 0.97 (0.06)<br>AC % 2-back post: 0.82 (0.17)	50	N-back task (1-back and 2-back)
Nouchi, 2014	DS-F post: 5.93 (1.19)<br>DS-B post: 4.60 (1.58)	30	DS-F post: 5.71 (1.02)<br>DS-B post: 4.71 (1.51)	31	Digit Span Forward<br>Digit Span Backward
Vaughn, 2014	LNS post: 16.3 (3.8)	25	LNS post: 15.2 (3)	23	Letter-Number Sequencing
Bantoft, 2015	DS-F (walk): 11.73 (2.20)<br>DS-B (walk): 7.28 (2.56)<br>LNS (walk): 11.47 (2.55)	45	DS-F (sit): 11.40 (2.24)<br>DS-B (sit): 7.22 (1.98)<br>LNS (sit): 11.49 (2.35)	45	WAIS-III Digit Span Forward WAIS-III Digit Span Backward<br>WAIS-III Letter-Number Sequencing
Basso, 2015[2]	DST post: 20.16 (2.328)	43	DST post: 19.90 (2.186)	42	Digit Span Forward<br>Digit Span Backward
Howie, 2015[1]	DS 10 min: 18.2 (4.85)	94	DS 10 min: 17.8 (4.85)	94	Digit Span modified
Albinet, 2016	Correct N-back post: 26.2 (1.8)<br>Spatial post: 26.6 (7.7)<br>Verbal post: 37.8 (7.8)	19	Correct N-back post: 25.4 (2.2)<br>Spatial post: 23.4 (12)<br>Verbal post: 34.3 (9.5)	17	N-back task (2-back)<br>Spatial Running Span task<br>Verbal Running Span task
Chapman, 2016[2]	RST post: 2.765 (0.865)	18	RST post: 3.211 (0.819)	19	Reading Span Task (Daneman Carpenter)

Table S1. Supplementary data regarding post-intervention scores for all included studies.

[1]Standard Errors reported which were transformed to Standard Deviations via Cochrane recommended methodology

[2]All DST scores were averaged into one score
