# Supplementary material for: The effects of chronic and acute physical activity on working memory performance in healthy participants: a systematic review with meta-analysis of randomized controlled trials
Source: Syst Rev. 2017 Jun 30;6:124. doi: 10.1186/s13643-017-0514-7 (PMC5493123; doi:10.1186/s13643-017-0514-7)
Supplement: Supplementary file 5 — Moderation analyses. (PDF 226 kb) [file 13643_2017_514_MOESM5_ESM.pdf]

Groups		Effect size and 95% confidence interval					Test of null (2-Tail)		Heterogeneity				Tau-squared			
Group	Number Studies	Point estimate	Standard error	Variance	Lower limit	Upper limit	Z-value	P-value	Q-value	df (Q)	P-value	I-squared	Tau Squared	Standard Error	Variance	Tau
**Fixed effect analysis**																
18-64 yo	3	0.064	0.086	0.007	-0.105	0.233	0.741	0.459	5.122	2	0.077	60.955	0.042	0.075	0.006	0.206
5-17 yo	9	0.006	0.068	0.005	-0.127	0.140	0.090	0.928	3.784	8	0.876	0.000	0.000	0.023	0.001	0.000
65 yo and	5	0.324	0.071	0.005	0.185	0.463	4.565	0.000	3.915	4	0.418	0.000	0.000	0.020	0.000	0.000
Total within									12.821	14	0.541					
Total between									11.353	2	0.003					
Overall	17	0.135	0.043	0.002	0.052	0.219	3.169	0.002	24.174	16	0.086	33.813	0.016	0.018	0.000	0.128
**Mixed effects analysis**																
18-64 yo	3	0.016	0.157	0.025	-0.292	0.323	0.100	0.920								
5-17 yo	9	0.006	0.068	0.005	-0.127	0.140	0.090	0.928								
65 yo and	5	0.324	0.071	0.005	0.185	0.463	4.565	0.000								
Total between									11.202	2	0.004					
Overall	17	0.146	0.047	0.002	0.054	0.238	3.107	0.002								

Figure S1. Moderation analysis of age according to WHO global recommendations on physical activity for health by age.

Groups		Effect size and 95% confidence interval					Test of null (2-Tail)		Heterogeneity				Tau-squared			
Group	Number Studies	Point estimate	Standard error	Variance	Lower limit	Upper limit	Z-value	P-value	Q-value	df (Q)	P-value	I-squared	Tau Squared	Standard Error	Variance	Tau
**Fixed effect analysis**																
Low	5	0.273	0.062	0.004	0.152	0.394	4.420	0.000	6.451	4	0.168	37.994	0.012	0.023	0.001	0.111
Moderate	3	-0.055	0.094	0.009	-0.239	0.129	-0.586	0.558	0.737	2	0.692	0.000	0.000	0.034	0.001	0.000
Moderate to	9	0.051	0.076	0.006	-0.098	0.201	0.676	0.499	6.696	8	0.570	0.000	0.000	0.028	0.001	0.000
Total within									13.884	14	0.458					
Total between									10.290	2	0.006					
Overall	17	0.135	0.043	0.002	0.052	0.219	3.169	0.002	24.174	16	0.086	33.813	0.016	0.018	0.000	0.128
**Mixed effects analysis**																
Low	5	0.269	0.082	0.007	0.109	0.430	3.288	0.001								
Moderate	3	-0.055	0.094	0.009	-0.239	0.129	-0.586	0.558								
Moderate to	9	0.051	0.076	0.006	-0.098	0.201	0.676	0.499								
Total between									7.399	2	0.025					
Overall	17	0.098	0.048	0.002	0.004	0.192	2.053	0.040								

Figure S2. Moderation analysis of physical activity intensity. Basso *et al.* 2015 which had a 'vigorous' intensity was grouped with 'moderate to vigorous' for purpose of practical analysis.

Groups		Effect size and 95% confidence interval					Test of null (2-Tail)		Heterogeneity				Tau-squared			
Group	Number Studies	Point estimate	Standard error	Variance	Lower limit	Upper limit	Z-value	P-value	Q-value	df (Q)	P-value	I-squared	Tau Squared	Standard Error	Variance	Tau
**Fixed effect analysis**																
Unknown	11	0.091	0.050	0.003	-0.008	0.190	1.794	0.073	11.643	10	0.310	14.115	0.005	0.015	0.000	0.069
Yes	6	0.249	0.080	0.006	0.091	0.406	3.099	0.002	9.752	5	0.083	48.730	0.047	0.066	0.004	0.218
Total within									21.396	15	0.125					
Total between									2.778	1	0.096					
Overall	17	0.135	0.043	0.002	0.052	0.219	3.169	0.002	24.174	16	0.086	33.813	0.016	0.018	0.000	0.128
**Mixed effects analysis**																
Unknown	11	0.089	0.056	0.003	-0.020	0.199	1.597	0.110								
Yes	6	0.138	0.134	0.018	-0.124	0.400	1.032	0.302								
Total between									0.114	1	0.736					
Overall	17	0.096	0.052	0.003	-0.005	0.197	1.871	0.061								

Figure S3. Moderation analysis of allocation concealment.

Groups		Effect size and 95% confidence interval					Test of null (2-Tail)		Heterogeneity				Tau-squared			
Group	Number Studies	Point estimate	Standard error	Variance	Lower limit	Upper limit	Z-value	P-value	Q-value	df (Q)	P-value	I-squared	Tau Squared	Standard Error	Variance	Tau
**Fixed effect analysis**																
No	3	0.085	0.108	0.012	-0.127	0.297	0.790	0.430	4.789	2	0.091	58.241	0.053	0.093	0.009	0.231
Unknown	10	0.085	0.056	0.003	-0.024	0.195	1.528	0.127	7.181	9	0.618	0.000	0.000	0.016	0.000	0.000
Yes	4	0.279	0.084	0.007	0.114	0.443	3.318	0.001	8.281	3	0.041	63.771	0.064	0.088	0.008	0.254
Total within									20.251	14	0.122					
Total between									3.923	2	0.141					
Overall	17	0.135	0.043	0.002	0.052	0.219	3.169	0.002	24.174	16	0.086	33.813	0.016	0.018	0.000	0.128
**Mixed effects analysis**																
No	3	0.029	0.175	0.031	-0.314	0.373	0.168	0.866								
Unknown	10	0.085	0.056	0.003	-0.024	0.195	1.528	0.127								
Yes	4	0.175	0.163	0.027	-0.144	0.494	1.076	0.282								
Total between									0.401	2	0.818					
Overall	17	0.089	0.051	0.003	-0.010	0.189	1.767	0.077								

Figure S4. Moderation analysis for the presence of blinding of either participants or personnel.

Groups		Effect size and 95% confidence interval					Test of null (2-Tail)		Heterogeneity				Tau-squared			
Group	Number Studies	Point estimate	Standard error	Variance	Lower limit	Upper limit	Z-value	P-value	Q-value	df (Q)	P-value	I-squared	Tau Squared	Standard Error	Variance	Tau
**Fixed effect analysis**																
Acute	9	0.019	0.061	0.004	-0.100	0.138	0.317	0.752	2.826	8	0.945	0.000	0.000	0.018	0.000	0.000
Chronic	8	0.251	0.060	0.004	0.132	0.369	4.156	0.000	14.018	7	0.051	50.064	0.031	0.035	0.001	0.177
Total within									16.844	15	0.328					
Total between									7.330	1	0.007					
Overall	17	0.135	0.043	0.002	0.052	0.219	3.169	0.002	24.174	16	0.086	33.813	0.016	0.018	0.000	0.128
**Mixed effects analysis**																
Acute	9	0.019	0.061	0.004	-0.100	0.138	0.317	0.752								
Chronic	8	0.190	0.092	0.009	0.009	0.370	2.055	0.040								
Total between									2.384	1	0.123					
Overall	17	0.070	0.051	0.003	-0.029	0.170	1.393	0.164								

Figure S5. Moderation analysis of intervention length of the included physical activity studies.
